# Azithromycin through the Lens of the COVID-19 Treatment

**DOI:** 10.3390/antibiotics11081063

**Published:** 2022-08-05

**Authors:** Georgia G. Kournoutou, George Dinos

**Affiliations:** Department of Biochemistry, School of Medicine, University of Patras, 26504 Patras, Greece

**Keywords:** macrolides, azithromycin, virus, coronavirus, COVID-19, immunolides, antivirus

## Abstract

Azithromycin has become famous in the last two years, not for its main antimicrobial effect, but for its potential use as a therapeutic agent for COVID-19 infection. Initially, there were some promising results that supported its use, but it has become clear that scientific results are insufficient to support such a positive assessment. In this review we will present all the literature data concerning the activity of azithromycin as an antimicrobial, an anti-inflammatory, or an antivirus agent. Our aim is to conclude whether its selection should remain as a valuable antivirus agent or if its use simply has an indirect therapeutic contribution due to its antimicrobial and/or immunomodulatory activity, and therefore, if its further use for COVID-19 treatment should be interrupted. This halt will prevent further antibiotic resistance expansion and will keep azithromycin as a valuable anti-infective therapeutic agent.

## 1. Introduction

**Figure 1:**
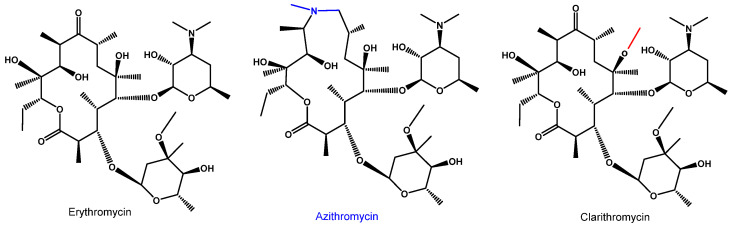
Molecular structure of the mother macrolide molecule erythromycin and its semisynthetic derivatives azithromycin (15-membered) and clarithromycin (14-membered). Blue and red colors in the structures represent modifications of the mother molecule (black).

Azithromycin (Azi) belongs to the large family of macrolide antibiotics, an important class of first-line antimicrobial agents [1]. Azi belongs to the second generation of macrolides, as a semisynthetic derivative of erythromycin with a modified macrolactone ring with 15 members instead of 14 members as in erythromycin (Figure 1). Although Azi did not exhibit improved activity against Gram-positive bacteria compared to the mother compound erythromycin [2,3,4], it was selected for further development due to its enhanced pharmacokinetic profiles. In particular, it was selected for its high half-life time and the ability to accumulate at high levels within lung tissue [5,6,7,8,9]. Clarithromycin (Figure 1) is another key second-generation 14-membered macrolide with similar features and structure, and while it was initially included in a few trial schemes as a potential therapeutic drug for COVID-19, it was rapidly discontinued [10,11]. Azi, like most of the other macrolides, is not only known for its antimicrobial activity, but it also has additional actions as either anti-inflammatory or antivirus agents. In this review we will present a summary of the existing literature data concerning azithromycin and will explain why it was initially hypothesized to have activity for COVID-19 treatment. Additionally, all studies on the use or non-use of azithromycin in the treatment of COVID-19 will be presented. Finally, we will discuss why Azi is not included anymore in therapeutic protocols and why its use must be interrupted to avoid increasing Azi pathogen resistance thereby maintaining the antibiotic as a useful therapeutic weapon for a longer time.

## 2. Methods

The authors searched the PubMed and the Scopus database using the terms “Coronavirus”, “COVID-19”, and “Azithromycin”. The same search terms were used for searching the Clinical Trials database. The abstracts were screened and only the relevant articles were considered in the review. Articles from the Clinical Trials database that were limited by a small sample size or other criteria were declared not relevant, and therefore, were not considered. Articles published as recently as 15 April 2022 were included.

## 3. Azithromycin as an Antimicrobial Agent

**Figure 2:**
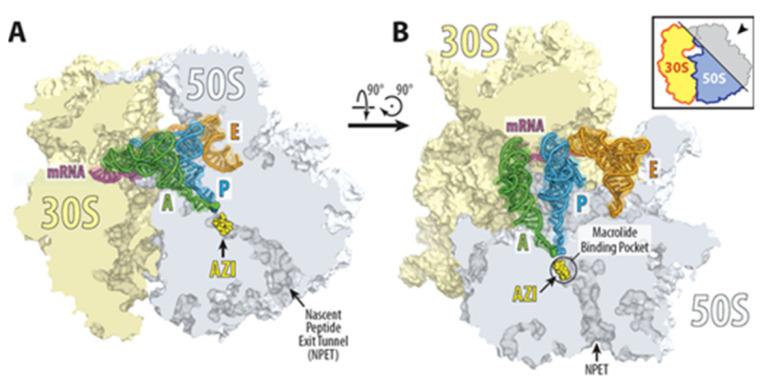
Structure of azithromycin in complex with the 70S ribosome carrying A-, P-, and E-site tRNAs. (**A**,**B**) Location of the ribosome-bound azithromycin (yellow) in the macrolide binding pocket at the entrance to the nascent peptide exit tunnel (NPET) of the 70S ribosome relative to tRNAs viewed as cross-cut sections through the ribosome. The 30S subunit is shown in light yellow, the 50S subunit is in light blue, the mRNA is in magenta, and the A-, P-, and E-site tRNAs are colored green, dark blue, and orange, respectively. The phenylalanyl and formyl-methionyl moieties of the A- and P-site tRNAs are shown as spheres [15].

The antimicrobial activity of azithromycin results from its binding with high affinity to the entrance of the ribosomal exit tunnel of prokaryotic 70S ribosomes and strongly inhibiting the bacterial protein synthesis [12,13,14]. According to the crystal structure data, it binds to the entrance of the nascent peptide exit tunnel and partially occludes it (Figure 2).

Thus, Azi was considered as ‘tunnel plugs’ that inhibit the synthesis of every protein entering the exit tunnel [1,16]. However, more recent evidence demonstrates that macrolides selectively inhibit the translation of a subset of cellular proteins and that their action crucially depends on the nascent protein sequence and the antibiotic structure [17].

Recent studies have shown that the translation of many genes was arrested at a few distinct sites through the length of the gene after treatment with macrolide antibiotics. Analysis of the sites of the stops revealed the existence of specific sequence signatures that induce pronounced drug-induced translation arrest and lead to specific regulation of protein synthesis. Ribo-seq and toeprinting experiments have revealed leader ORFs of macrolide resistance genes carrying the +x+ motif, where + stands for positively charged amino acids lysine or arginine, and x stands for any amino acid [17,18].

Therefore, Azi emerges as a modulator of translation rather than as a global inhibitor of protein synthesis. In general, macrolide antibiotics are active mainly against Gram-positive bacteria and have a lower activity against Gram-negative bacteria [19,20]. Macrolides are very active against Gram-positive bacteria *Staphylococcus*, *Streptococcus*, and *Diplococcus*; among Gram-negative cocci, *Neisseria gonorrhea*, *Haemophilus influenzae*, *Bordetella pertussis,* and *Neisseria meningitis*; and are extremely active against various Mycoplasmas. Since its discovery Azi has been extensively used in the treatment of bacterial and mycobacterial infections of the respiratory, gastrointestinal, genitourinary, and cutaneous systems [21,22,23]. Azi is a member of the WHO list of essential medications [24] and is available in large quantities worldwide. Despite some mild side effects, including mainly diarrhea and QT prolongation, Azi is proven to be safe and cheap, and therefore, easily available to humans worldwide [21,22].

## 4. Azithromycin as an Anti-Inflammatory Agent

**Figure 3:**
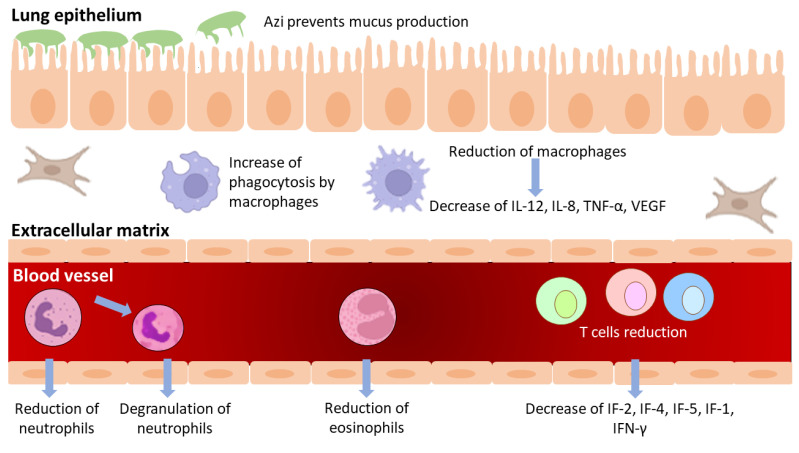
Immunomodulatory effects of azithromycin.

Beyond the antibacterial activity of azithromycin, and broadly most macrolides, their anti-inflammatory effects have been established and some of them have been used in chronic inflammatory diseases such as chronic rhinosinusitis, bronchial asthma, bronchiectasis, chronic obstructive pulmonary disease, cystic fibrosis, etc. [23,24,25,26,27]. Probably, the most striking example of their immunomodulation comes from diffuse panbronchiolitis, an idiopathic inflammation and progressively destructive disease of the bronchioles which can be converted from a lethal to a treatable disease with daily low-dose erythromycin or Azi [23,28,29]. This has been accredited to the ability of Azi to normalize the upregulated activities of IL-1β, IL-2, TNF, and GM-CSF [30]. Azi is rapidly absorbed after oral administration with a half-life time of approximately 3 days, leading to a high and constant tissue concentration [23,31]. As a result, Azi accumulates in human cells, including epithelial cells, and most notably in phagocytes where it has been concentrated hundreds to thousands of times with a focus on phagocyte lysosomes [9,31]. Its anti-inflammatory or immunomodulatory activity reported in several studies includes the most frequent effects on neutrophils, monocytes, and lymphocytes [27,29,32]. Among the usually measured immunological modified markers are the number of decreased neutrophils; the concentrations of neutrophil elastase; cytokines release; surface-expressed molecules (mainly Toll-like receptors); superoxide production; and cell homeostasis, mainly apoptosis and phagocytosis (Figure 3) [27,29,32,33]. Neutrophil function inhibition has been reported more frequently than eosinophil function inhibition. Azi stimulates neutrophil degranulation and phagocytosis-associated oxidative burst, mediated via modulation of ERK 1/2 signaling [19]. These initial stimulatory effects are followed by modulation of transcription factors activator protein (AP)-1, nuclear factor kappa B (NFκB), inflammatory cytokines, and mucin release, with overall anti-inflammatory effects [34].

Azi inhibits lipopolysaccharide-induced pro-inflammatory cytokines; increases phagocytosis by inhibiting AP-1 [35]; improves lysosomal resistance to oxidant challenge [36]; and promotes M2 polarization of macrophages (a process in which macrophages produce distinct functional phenotypes in response to specific microenvironmental stimuli and signals) [37,38,39]. Azi can also increase the phagocytosis of apoptotic epithelial cells [40] and neutrophils by macrophages [41] further supporting its anti-inflammatory activity. Studies have shown that part of the immunomodulatory effects of macrolides could be attributed to the impairment of TLR signaling by reducing the release of PAMPs (Pathogen-Associated Molecular Patterns) and inhibiting TLR expression, either of dendritic cells or macrophages, thereby regulating the immune response [33,42].

Immunomodulatory effects, although similar to most therapeutic macrolides, are likely to differ among them. Few studies have examined the anti-inflammatory effects of macrolides on more than one macrolide, and none of the human trials have explicitly compared different macrolides. Furthermore, the majority of these trials were conducted on healthy volunteers and/or Azi was administered in varying doses at a time [27,43,44,45]. Clinical investigations in CF patients, on the other hand, revealed that Azi, but not Clarithromycin, improves respiratory function and reduces pulmonary exacerbations [46,47]. Additionally, another study showed that Azi, but not clarithromycin or roxithromycin, inhibits IL-1alpha and IL-1beta production [48]. In general, azithromycin inhibits the synthesis of pro-inflammatory cytokines by both innate and adaptive immune cells, as well as the accumulation, adhesion, and death of pulmonary neutrophils [32].

Azithromycin, like other macrolides, has very low activity against eukaryotes due to their low affinity for binding to eukaryotic ribosomes [1]. There are specific differences between eukaryotic and bacterial ribosomes (differences between rRNA bases or ribosomal proteins) that mediate the selectivity and toxicity of ribosomal drugs, as established by rRNA sequencing studies and X-ray crystallography [49].

## 5. Azithromycin as an Antivirus Agent

Azi’s antiviral effects have been demonstrated in vitro, albeit not all examples have been confirmed in vivo [32]. Since Azi mediated exacerbations in airway diseases, particularly in asthma [25,50], its effects were studied against viruses that cause such airway infections such as rhinoviruses (RV). Azi inhibits RV replication and releases in primary human bronchial epithelial cells in vitro [51]. The AMAZES research, the largest clinical trial of a long-term macrolide on airway diseases, found that Azi reduced asthma exacerbations by 40% in vivo [25]. The mechanism is not known, but metagenomic analysis suggested that it could be related to an antibacterial effect versus *Haemophilus influenzae* and possibly its abundance in inhaled air [52,53,54]. Pre-treatment with azithromycin inhibits RV replication in CF bronchial epithelial cells, probably by amplifying the antiviral response mediated by the IFN pathway [55]. Additionally, Azi showed a reduction in H1N1 viral replication in A549 cells with IC_50_ 58 μM interfering with the internalization of viruses [56]. In experiments with the Zika virus, within glial cell lines and human astrocytes, there was a reduction in viral growth and virus-induced cytotoxicity [57]. Equally, Azi inhibited Ebola replication with EC_50_ 5.1 μM and low toxicity; however, it did not boost survival in mice or guinea pigs when tested in vivo in a mouse model [58].

The precise mechanism of the antiviral activity of Azi remains unclear. Given that Azi is a weak base it can accumulate in acidic intracellular organelles such as endosomal vesicles and lysosomes [59]. In keeping with lysosomal accumulation, azithromycin causes lysosomal pH change [23]. This modified acidic environment caused by accumulation of Azi could also be responsible for uncoating enveloped viruses such as influenza and maybe coronavirus [59]. Data also suggest that the antiviral activity of Azi could be attributed to its ability to increase the expression of the epithelial interferon genes, leading to a reduction in viral replication [60].

Recently, Azi and spiramycin (a natural 16-membered ring macrolide) provided significant in vivo protection against enterovirus-A71 infection in mice [61]. Spiramycin was found to interfere with EV-A71 viral RNA synthesis, and it is likely that spiramycin and Azi function in concert after the viral entrance; thereby, inhibiting viral RNA synthesis either directly or indirectly.

## 6. Azithromycin and Betacoronovirus

From the beginning of the current SARS-CoV-2 pandemic, several drug screens were conducted in a rapid, urgent manner to evaluate potential candidate medications against this pathogen. The requirements were: to be approved, to be inexpensive, to be safe, and to be available as quickly as was feasible worldwide. Previous screens had recognized more than 90 drugs that inhibited SARS-CoV-2 viral replication with EC_50_ nearly to 10 μM [62]. The tested drugs included protease inhibitors, ATPase proton pump inhibitors, viral protease inhibitors, compounds targeting the angiotensin pathway, and antibiotics. Azi tested in Vero E6 cells had an EC_50_ of 2.12 μM and EC_90_ equal to 8.65 μM, and selectivity index >19 [63], which is very comparable to the control antiviral-compound remdesivir (EC_50_ = 1.65, EC_90_ = 2.52), the first antiviral agent with proven clinical efficacy against SARS-CoV-2 in all clinical trials [64,65,66]. Azi was also discovered as a target in a bioinformatic screening investigation of potentially relevant pathways that may be turned into pharmaceutically acceptable forms. An initial study focused on two of the previous candidate molecules, hydroxychloroquine (HQL) and Azi, suggested a synergistic inhibition of SARS-CoV-2 replication in Vero cells at 5 and 10 μM concentrations, respectively [67,68]. This synergy was presented as a way to make hydroxychloroquine more effective at less hazardous concentrations. It was the first observational study suggesting that HQL, especially when combined with Azi, improved virological clearance [69]. However, because the data with Azi came from only six patients and the study was open-label and nonrandomized, no acceptable conclusions could be derived statistically [70]. This Azi-HQL combination was also investigated in nonhuman primates, but no substantial antiviral effect was observed in the five macaques given Azi in addition to hydroxychloroquine [71]. Furthermore, this initial favorable finding led to the immediate start of interventional trials to assess the efficacy of the COVID-19 therapy combination, as well as the efficacy of Azi with HQL. Hundreds of trials with Azi are listed on clinical trials.gov. Initially, Azi was prescribed as an adjunct to hydroxychloroquine, but later HQL was largely abandoned and Azi was used alone. From the beginning of 2020, decades of publications were released either favoring or discouraging the use of Azi, both with or without HQL [70]. According to them, Azi was initially favored with or without HQL [72,73,74,75,76,77] but at the same time more observations did not favor its use [78,79,80,81,82,83,84,85,86]. Since most of them were retrospective studies, it was clear that randomized control trials (RCTs) were necessary to clarify the previous controversial data. All these RCTs were integrated during the previous year and are presented in Table 1. The table gives an overview of the most currently published, up-to-date, peer-reviewed studies in the literature, in which the effect of Azi is evaluated. Although these RCTs in Table 1 differ in their outcomes and whether or not hospitalized patients are included, all of them suggested that azithromycin does not reduce hospital admissions, respiratory failure, or death when compared to conventional therapy, and therefore, Azi should no longer be used to treat COVID-19. In a few words, all of them showed that, in hospitalized patients with COVID-19, azithromycin did not reduce the time to sustained clinical improvement or discharge. There is clearly no efficacy in terms of clinical status or mortality at the fixed time points used in all scientifically acceptable large trials.

Furthermore, according to Oldenburg et al., there was no significant difference in self-reported symptom absence 14 days after enrollment among patients assigned to azithromycin versus a placebo in their randomized controlled trial of single-dose oral azithromycin for outpatient COVID-19 [11]. This last finding supports earlier randomized clinical trials of azithromycin for COVID-19 in both outpatient and inpatient settings, none of which found azithromycin to be effective in treating COVID-19.

Given that azithromycin consumption during the pandemic was increased up to 3 times compared to the pre COVID period [91,92,93], it is important to reduce useless consumption, as it is an extremely dangerous practice, to avoid increasing antimicrobial resistance (AMR). Antimicrobial resistance (AMR) develops when bacteria, fungi, or viruses are exposed to antibiotics, antifungals, or antivirals leading to the development of a resistance to one or more antimicrobial drugs. As a result, the antimicrobials become ineffective and infections may persist. AMR is considered a serious and persistent therapeutic problem today being an economic and health burden. It is conservatively estimated that, in the US and Europe, 2.5 million people are affected by such infections each year and approximately 50,000 people die because of these infections [94]. The discovery of novel antibiotics has nearly halted over the past 30 years leading to the exhaustion of the pipeline reserve. The resistance of pathogens to antibiotics can be addressed with a rapid development of new effective and safe antibiotics [1,95]. Several studies have revealed a significant increase in drug resistance to azithromycin in some strains of *gonococci* [96]. Drug resistance to azithromycin is also increasing in *E. coli* [95].

Identifying strategies that can work to reduce the burden of bacterial AMR—either across a wide range of settings or those that are specifically tailored to the resources available and leading pathogen–drug combinations in a particular setting—is an urgent priority [97]. Since the prevalence of bacterial superinfection in COVID-19 is low [98], and unlike influenza [99,100,101], there is no preventive benefit against postviral pneumococcal and atypical pneumonia [98], it will be extremely helpful to avoid the useless consumption of any antibiotic prescription, specifically azithromycin, in COVID-19 treatment..

The outbreak of a pandemic led to a massive disruption of healthcare systems which overshadowed the misuse and incorrect prescription of some antibiotics [102]. Many COVID-19 patients received empirical antibiotic therapy for COVID 19 treatment in the early stages of pandemic since it was considered the safer option due to clinical uncertainty [93]. Control policies should be administrated in clinical practice regarding the use of drugs in the treatment of COVID-19. As AMR will be a major clinical problem, stewardship activities are necessary in the coming years to face the new-pandemic [102].

## 7. Closing Remarks and Perspectives

During the pandemic of coronavirus, antibiotics prescription was elevated without justification, partly because the medical community was unprepared for this burst and secondly because the clinical situation of patients changed dramatically each day after the initial day of infection. The administration of known antibiotics was considered to be the correct way of combating coronavirus but it soon became clear that there was no justification for the overuse of antibiotics as they did not decrease the risk of mortality in the patients who had no reason to receive this treatment [103].

To summarize, there is no scientific justification for the use of azithromycin in the treatment of COVID-19 up to now, and the only way to keep this antibiotic relevant in the future as a useful tool for combating pathogenic infections is to use it wisely, only after careful consideration and high expectations.

## Figures and Tables

**Table 1:** Published RCTs assessing the effect of Azithromycin on COVID-19 treatment.

Name of Clinical Trial	Year	Participants	Clinical Outcome	Type of Study
Furtado et al., 2020 (COALITION II)[87]	2020	447	No justification for the use of azithromycin for better clinical outcomes.	Open label
Butler et al., 2021 (PRINCIPLE)[88]	2021	292	No justification for the use of azithromycin for reducing time to recovery or risk of hospitalization.	Open label
Hinks et al., 2021 (ATOMIC 2)[89]	2021	263	No justification for the use of azithromycin for reducing the risk of hospitalization or death.	Open label
Horby et al., 2021 (RECOVERY) [90]	2021	2265	No justification for the use of azithromycin on inpatients for increase in survival.	Open label
Oldenburg et al., 2021 [11]	2021	7763	No justification for the use of azithromycin versus placebo for elimination of symptoms at day 14.	Blind
Gyselinck et al., 2022 (DAWn-AZITHRO) [70]	2022	160	Early trial termination, failed to demonstrate a benefit of azithromycin.	Open label

## Data Availability

All relevant data are presented in the article.

## Abbreviations

Azi: azithromycin; IL-1β: interleukin 1β; IL-2: interleukin 2; TNF: tissue necrosis factor; GM-CSF: granulocyte macrophage colony stimulating factor; TLR: Toll-like receptor; AP-1: activator protein-1; EV-A71: enterovirus A71; RV: rhinovirus; CF; cystic fibrosis; IFN: interferon; HQL: hydroxychloroquine; ERK1/2: extracellular signal related regulated kinase 1/2.
